# Dimethyl Fumarate Prevents the Development of Chronic Social Stress-Induced Hypertension in Borderline Hypertensive Rats

**DOI:** 10.3390/antiox13080947

**Published:** 2024-08-03

**Authors:** Michal Kluknavsky, Peter Balis, Silvia Liskova, Andrea Micurova, Martin Skratek, Jan Manka, Iveta Bernatova

**Affiliations:** 1 Centre of Experimental Medicine, Institute of Normal and Pathological Physiology, Slovak Academy of Sciences, 813 71 Bratislava, Slovakia; michal.kluknavsky@savba.sk (M.K.); peter.balis@savba.sk (P.B.); silvia.liskova@fmed.uniba.sk (S.L.); andrea.micurova@savba.sk (A.M.); 2 Institute of Pharmacology and Clinical Pharmacology, Faculty of Medicine, Comenius University, 811 08 Bratislava, Slovakia; 3 Institute of Measurement Science, Slovak Academy of Sciences, 841 04 Bratislava, Slovakia; martin.skratek@savba.sk (M.S.); jan.manka@savba.sk (J.M.)

**Keywords:** NRF2, *Nfe2l2*, chronic stress, cardiovascular function

## Abstract

This study investigated the effects of chronic crowding-induced social stress and dimethyl fumarate (DMF) on borderline hypertensive rats, focusing on the transcription nuclear factor (erythroid-derived 2)-like 2 (NRF2) gene *Nfe2l2*, on the expression of selected NFR2-mediated gene expressions in the heart, and on vascular function. Rats were exposed to chronic crowding, DMF treatment (30 mg/kg/day, p.o.), or a combination of both for six weeks. Blood pressure (BP) was measured non-invasively, gene expressions were analysed using RT-qPCR, and vascular function was assessed by measuring noradrenaline (NA)-induced vasoconstriction and endothelium-dependent and -independent relaxations in the femoral arteries using a wire myograph. Chronic stress increased BP, *Nfe2l2* expression, and NA-induced vasoconstriction, though it did not affect relaxation responses nor the left heart ventricle-to-body weight (LHV/BW) ratio. DMF elevated *Nfe2l2* expression (as the main effect) in the heart but did not alter BP and vascular functions vs. control when administered alone. Interestingly, DMF increased the LHV/BW ratio, supposedly due to reductive stress induced by continuous NRF2 activation. When combined with stress, DMF treatment prevented stress-induced hypertension and mitigated NA-induced vasoconstriction without altering relaxation functions. In addition, the combination of stress and DMF increased *Tnf* and *Nos2* expression and the expressions of several genes involved in iron metabolism. In conclusion, these findings suggest that DMF can prevent chronic stress-induced hypertension by reducing vascular contractility. Moreover, DMF itself may produce reductive stress in the heart and induce inflammation when combined with stress. This indicates a need for the careful consideration of long-term DMF treatment considering its impact on the heart.

## 1. Introduction

Cardiovascular diseases (CVDs), including hypertension, are among the leading causes of death worldwide. Prehypertension is a condition in which blood pressure (BP) is elevated above normal levels but not yet high enough to be classified as a diseased state. It is a relatively common condition even in children and young adults [1,2]. Chronic stress is considered to be an etiological factor in the development of hypertension in humans [3,4] in which (psycho)social stress is the dominant source of negative impact on health and wellbeing. Stress can further accelerate the development of hypertension in prehypertensive individuals with a significantly increased risk of target organ damage [4,5]. In addition to well-known hormonal and neurohumoral alterations aiming to maintain cardiovascular homeostasis, stress results in the mobilisation of energetic sources, alterations in iron metabolism, and induction of inflammatory responses [6,7].

The master regulator of cytoprotective responses that play a significant role in the protection against the development of non-communicative diseases is the transcription nuclear factor (erythroid-derived 2)-like 2 (NRF2) [8]. NRF2 plays a key role in cytoprotection and protection against low-grade oxidative stress and inflammation in the cardiovascular system (CVS). In addition to Kelch-like ECH-associated protein 1 (KEAP1), NRF2 was shown to be regulated at the transcriptional level [9]. NRF2 is known to upregulate the expression of antioxidant enzymes and anti-inflammatory factors, reduce the expression of inflammatory mediators, and also participate in the regulation of iron metabolism [8].

Regarding the intracellular homeostasis of iron, which is regulated at the transcriptional level [10], several experimental studies have shown altered levels of non-heme iron and expressions of ferroportin (FPN1), ferritin (FTH1), and transferrin receptor 1 (TFR1) genes (*Fpn1*, *Fth1*, and *Tfr1*) upon NRF2 activation/inactivation [11,12]. In addition, several experimental studies investigating the effects of various stressors have described a decrease in serum iron, haemoglobin, FTH1, increased hepatic iron content, and reduced protein expression of the iron exporter FPN1 in the liver [13]. However, only a few studies have investigated the effects of NRF2 activation/inactivation on iron metabolism in the heart. The associations between iron levels and the incidence of prehypertension and hypertension were found in children and adolescents [14]. In preclinical studies, the relationship between iron metabolism, NFR2, and CVDs was shown using Nrf2^−/−^ mice which develop age-dependent cardiomyopathy, characterised by iron accumulation and severe protein oxidation. In addition, dietary iron deficiency increased heart weight, increased the accumulation of carbonylated proteins, and induced the nuclear translocation of NRF2 [15,16].

BP is a complex parameter which is affected by heart function and the function of the vascular bed and regulated by a complex network of the central and autonomic nervous systems and neurohumoral factors [17,18]. An important factor in maintaining BP is nitric oxide (NO), which affects BP via the regulation of endothelium-dependent relaxation and acts as a neurotransmitter and neuromodulator in the central and peripheral nervous system. Stress alters NO bioavailability in the vasculature, heart, and other organs, which all participate in the systemic regulation of BP [19]. Pivotal mechanisms in the pathogenesis of elevated BP are associated with oxidative stress and chronic inflammation, which can be accentuated in the presence of systemic stress and high intracellular iron. An increase in NO bioavailability due to the suppression of reactive oxygen species (ROS) formation through NRF2 activation may play a fundamental role in preventing endothelial dysfunction and hypertension development.

NRF2 function can be activated by various synthetic and natural substances such as sulforaphane, resveratrol, quercetin, bardoxolone methyl, and even registered drugs such as metformin and statins [9]. One of the most powerful NRF2 activators is dimethyl fumarate (DMF), which is approved for the treatment of psoriasis and sclerosis multiplex. DMF also possesses the potential to be repurposed for cardiovascular diseases as a therapeutic agent in patients with atherosclerotic cardiovascular disease [9,20]. However, less is known about the long-term activation of NRF2 function by DMF treatment in the CVS, namely in chronic social stress conditions.

To investigate the influence of genetic predisposition to high blood pressure, coming from one of the parents, the offspring of spontaneously hypertensive dams and normotensive Wistar–Kyoto sires are used in biomedical research. Since the resting mean arterial pressure of adult offspring in the F1 generation is in the range of 130–150 millimeters of mercury (mmHg) [21,22,23], they are called borderline hypertensive rats (BHRs). Because the BP regulation of BHRs is sensitive to external factors, they represent a suitable experimental model for stress-induced hypertension [22,24,25]. By using the mentioned rat model of prehypertension, it is possible to simulate the situation of a large part of the human population with a family history of hypertension.

Various rodent models of experimental stress can be used to investigate cardiovascular or neurobehavioral consequences of environmental stressors (such as restraint, immobilisation, shaking etc.) [26,27,28]. We use a model of crowding-induced stress that utilises continuous social interactions to evoke a stress response characterised by a significant psychosocial component [29]. While crowding itself is a mild stressor, chronic exposure can lead to neuroendocrine, behavioural, and cardiovascular changes in rats [23,29,30]. The relevance of this model to human situations was shown previously [31,32].

In this study, we examined the effects of the long-term administration of NRF2 activator DMF under normal and long-term crowding stress conditions in BHR. The study focused on investigating the expression of genes involved in antioxidant defence, inflammation, and iron metabolism in the heart. We tested the hypothesis that long-term DMF treatment would reduce the development of chronic social stress-induced hypertension in association with elevated *Nfe2l2* gene expression and increased expressions of selected genes involved in antioxidant defence, the reduced expression of genes involved in inflammation, and alterations in iron metabolism in the heart. Moreover, we hypothesised that the long-term administration of DMF may alter vascular functions in both control and chronic stress conditions.

## 2. Materials and Methods

### 2.1. Animals and Treatment

All procedures used in this study were approved by the Ethics Committee of the Centre of Experimental Medicine, Slovak Academy of Sciences, Bratislava, Slovakia and by the State Veterinary and Food Administration of the Slovak Republic, protocol code Ro-2654-3/2021-220 (from 17 February 2021) in line with the European Union Directive 2010/63/EU. All rats were bred in the accredited animal facility of the Centre of Experimental Medicine, Slovak Academy of Sciences, Bratislava, Slovakia, following the institutional guidelines. The animals were housed at constant humidity 45–65%, temperature 20–22 °C and 12 h dark/light cycle. All rats had ad libitum access to laboratory rat chow (Altromin 1324P, Altromin International, Lage, Germany). Ten-week-old BHR males, the offspring of SHR dams and WKY sires, were used in this study. The rats (n = 36) were divided into four groups: the control (Cont, n = 8), chronic crowding stress-exposed rats (Stress, n = 10), rats treated with DMF (DMF, n = 8), and rats exposed to the combined effect of stress and DMF (DMF+Stress, n = 10). The rats in the DMF and DMF+Stress groups were treated with DMF at a dose of approximately 30 mg/kg/day for six weeks by adding the DMF into drinking water. The daily drinking volume was assessed for each cage of rats before starting the experiment and adjusted daily. The DMF solution was prepared fresh every day before administration to rats by dilution of DMF in 0.5% dimethyl sulfoxide (DMSO) solution at room temperature for 30 min using a vortex. A calculated volume of DMF solution, based on the current body weight of the rats in the given cage and their 24 h drinking volume, was added to the drinking bottles to achieve the desired dose after all liquids had been consumed in 24 h. When the DMF solution was drunk earlier, the rats were given fresh water until the next dose of DMF was administered. The rats in both the Cont and Stress groups drank the vehicle (0.5% DMSO) solution using the same 24 h regime as when administering DMF. 

Crowding stress was induced by keeping the rats in a larger group per cage compared to the control group [23] with small modifications in the cage size. Rats housed in the control conditions (Cont and DMF groups, respectively) were kept at two rats per cage (39 cm width, 23.3 cm length, 23 cm height). Rats in the stress conditions (Stress and DMF+Stress, respectively) were kept at 5 rats per cage (39 cm width × 23.3 cm length × 23 cm height). Thus, crowding stress was induced by reducing the living space of rats to approximately 182 cm^2^/rat compared to 450 cm^2^/rat in the control group.

At the end of the experiment, rats were killed by decapitation after a short CO_2_ anaesthesia. One rat from each group was randomly excluded from this study and used for preliminary histological analyses. After decapitation, trunk blood was collected for further analysis. The heart, aorta and femoral arteries were dissected and the left heart ventricle (LHV) was separated and weighed. Subsequently, samples from the LHV, aorta, plasma, or blood were quickly sectioned/aliquoted for individual analyses, weighed and used as fresh samples, or frozen in liquid nitrogen and stored at −80 °C until further analyses.

### 2.2. Blood Pressure, Heart Rate, and Biometric Parameters Determination

Systolic blood pressure (BP) and heart rate (HR) were measured indirectly by tail-cuff plethysmography between 11.00 and 12.00 a.m., as was described in detail previously [33]. To minimise the influence of nonspecific stress on BP measurements, all rats were handled and accustomed to the tail-cuff procedure before experimentation in three independent sessions. Each BP value was calculated as the average of at least three measurements. BP and HR values were measured at the beginning of the experiment (Basal) and then weekly for six weeks. The body weight (BW) was determined after each BP measurement. BW gain was expressed as a percentage change from the basal BW value of each rat. The LHV-to-BW (LHV/BW) ratio was calculated as index of LHV hypertrophy.

### 2.3. Preparation of Blood Samples and Biochemical Analysis in Plasma

Trunk blood was collected into preprepared heparinised tubes (30 UI/1 mL) and then centrifuged (850× *g*, 10 min, 4 °C, Centrifuge 5430 R, Eppendorf, Hamburg, Germany). Plasma was stored at −80 °C until further analysis. Before the analysis, plasma samples were allowed to thaw to room temperature. Subsequently, 100 μL of plasma was pipetted into reagent discs for General Chemistry IV Lyophilised Kit and Clinical Emergency Lyophilised Kit (Celercare, MNCHIP Technologies, Tianjin, China) according to manufacturer protocol, and plasma parameters were determined by a biochemical analyser Celercare^®^ M5 (Celercare, MNCHIP Technologies, Tianjin, China).

### 2.4. Determination of Corticosterone

Plasma levels of corticosterone (pCort) were determined by a commercial colourimetric ELISA kit (ab108821, Abcam, Cambridge, UK). Plasma corticosterone was assayed in 25 μL of four-times-diluted plasma. Briefly, all reagents were equilibrated to room temperature and prepared per the manufacturer’s protocol. All standards, controls, and samples were assayed in duplicates. The samples were measured with a microplate reader (BioTek 800 TS, Ltd., Tianjin, China) at 450 nm.

### 2.5. Determination of Total Iron and Divalent Iron in Plasma

The levels of total iron, ferrous iron (Fe^2+^), and ferric iron (Fe^3+^) in plasma were determined by Iron Assay Kit according to the manufacturer’s protocols (ab83366, Abcam, Cambridge, UK). Briefly, 50 µL of the plasma sample was diluted 1:1 using Iron Assay Buffer (final volume 100 µL). Diluted plasma samples (100 µL) were used per well in a 96-well plate. The free Fe^2+^ reacted with the iron probe to produce a stable coloured complex. Then, the Fe^3+^ ions were reduced to Fe^2+^, enabling the measurement of total iron level. The samples were measured with a microplate reader (BioTek 800 TS, Tianjin, China) at 593 nm.

### 2.6. Gene Expression Determination

The gene expression levels of NRF2 gene *Nfe2l2*, superoxide dismutase 1 (*Sod1*), heme oxygenase 1 (*Hmox1*), glutathione peroxidase 4 (*Gpx4*), inducible nitric oxide synthase (NOS) (encoded by *Nos2*), endothelial NOS (*Nos3*), tumour necrosis factor alpha (*Tnf*), interleukin 1 beta (*Il1b*), transferrin receptor 1 (*Tfr1*), ferritin heavy chain 1 (*Fth1*), divalent metal ion transporter 1 (*Dmt1* or *Slc11a2*), ferroportin (*Fpn1*), and hepcidin (*Hamp*) were determined in the LHV by using real-time quantitative polymerase chain reaction (RT-qPCR). β-actin (*Actb*) was used as a housekeeping gene in the LHV. This suitable housekeeper gene was selected in a preliminary study, and its expression was not affected by stress, DMF, or their interaction.

The total RNA was isolated using the PureZOL™ RNA Isolation Reagent (Bio-Rad, Hercules, CA, USA), according to the manufacturer’s protocols. The amount and purity of total isolated RNA were spectrophotometrically quantified at 260/280 nm and 260/230 nm using a NanoDrop spectrophotometer (Thermo Scientific, Waltham, MA, USA). Reverse transcription was performed using 1 μg of total RNA from each sample. We used an Eppendorf Mastercycler (Eppendorf, Hamburg, Germany) and an iScript-Reverse Transcription Supermix (Bio-Rad, Hercules, CA, USA), following the manufacturer’s instructions [34]. The PubMed tool “Primer-BLAST” and “Gene” database were used to design gene-specific primers. In Table 1, the DNA sequences, melting temperature of the primers, the size of the amplicons, and the reference numbers of the templates are depicted. Data analysis has been performed using the 2^−ΔΔCT^ method [35].

### 2.7. Nitric Oxide Synthase Activity

Total NOS activity was determined in 20% of LHV and aorta homogenates (*w*:*v*) by measuring [^3^H]-L-citrulline formation from [^3^H]-L-arginine (MP Biochemicals, Santa Ana, CA, USA) as described previously [36]. The activity of L-citrulline formed at the enzymatic reaction was determined using the Quanta Smart Tri-Carb Liquid Scintillation Analyzer (PerkinElmer, Beaconsfield, UK). The results are expressed as picokatals per gram of protein (pkat/g protein). Protein content was determined by Lowry’s method.

### 2.8. Measurement of Conjugated Dienes

Conjugated dienes (CD), as a marker of lipid peroxidation and oxidative damage, were measured in 10% tissue homogenates (*w*:*v*) of the LHV. The exact methodological procedure for processing and isolating CD was described previously [37]. The absorbance of the samples was measured at 233 nm. To calculate the results, an extinction coefficient of 26,000 mol^−1^·L·cm^−1^ was used. The results were expressed in nanomoles of CD per gram of tissue (nmol/g).

### 2.9. Vascular Reactivity

Vascular parameters were determined in isolated and cleaned fresh femoral arteries with intact endothelium of Cont, DMF, Stress, and DMF+Stress groups (n = 7/group). Segments were placed in small-vessel dual-wire Mulvany–Halpern isometric myographs (Dual Wire Myograph system 520A, Multi Myograph System 620M, Danish Myo Technology A/S, Aarhus, Denmark), and the vascular reactivity was recorded using the LabChart 8 software package (ADInstruments, Oxford, UK).

In chambers, Krebs–Henseleit solution (in mmol/L: 119 NaCl, 4.7 KCl, 1.17 MgSO_4_·7H_2_O, 25 NaHCO_3_, 1.18 KH_2_PO_4_, 0.03 Na_2_EDTA, 2.5 CaCl_2_·2H_2_O, 5.5 glucose, 37 °C, pH 7.4) was used and bubbled with 95% O_2_ and 5% CO_2_. The inner arterial diameter of femoral arteries was normalised to 90% of the diameter predicted for the pressure of 100 mmHg. After 30 min of stabilisation, the viability of the isolated arteries was tested using a depolarising solution (125 mmol/L K^+^, NaCl was exchanged for an equimolar concentration of KCl) for 2 min. After being washed out, the experimental protocols were carried out as described in [33,38,39]. Briefly, ACh-induced endothelium-dependent relaxations were elicited on serotonin (10^−6^ mol/L) precontracted femoral arteries by cumulative application of acetylcholine (ACh, 10^−9^ to 10^−5^ mol/L). After washing, femoral arteries were precontracted by serotonin (10^−6^ mol/L) again, and the concentration–response curves on exogenous NO donor, sodium nitroprusside (SNP, 10^−9^ to 10^−5^ mol/L), were measured to determine the endothelium-independent relaxations. In another set of femoral arteries, the noradrenalin (NA)-induced concentration–response curves induced by noradrenalin (NA, Zentiva, Czech Republic) were elicited by cumulative concentrations of NA (10^−8^ to 10^−4^ mol/L).

All the chemicals were dissolved in distilled water. The concentrations were expressed as the final concentration in the myograph chamber. The results of ACh-induced and SNP-induced relaxations were expressed as the percentage of maximal precontraction achieved. Contractile functions were expressed as the wall tension in mN.

### 2.10. Determination of Magnetic Parameters

To determine the relative content of biogenic iron in LHV and blood, we used a Quantum Design MPMS-XL 7AC (SQUID) magnetometer (San Diego, CA, USA) with a reciprocating sample operation option with a sensitivity of 10^−11^ Am^2^ as described previously [40]. Before the analysis, LHV samples were defrosted and cut using the cylindrical instrument to repeatedly obtain the same shape of all samples with a diameter of ~4.5 mm. The samples were then vacuum-dried, weighed, and inserted into a plastic measuring straw. Blood (33 μL) was pipetted onto a pre-weighed 18 cm long and 6 mm narrow strip of standard office paper (80 g/m^2^) and was then dried in air for 24 h at room temperature, weighed, and inserted into a plastic measuring straw. For the determination of the relative content of biogenic iron, the magnetic characteristics of the samples were measured in the form of isothermal hysteresis curves at a temperature of 2 K (−271.15 °C) and a magnetic field up to 7 T to achieve the saturation magnetization (*Ms*). *Ms* is the parameter for determining the relative amount of magnetic compounds in the biological samples, of which iron is a dominant component [40]. Remanent magnetization (*Mr*) is the residual magnetization that remains after the removal of a magnetic field sufficient to reach *Ms*. Magnetic coercivity (*Hc*) is the intensity of the applied magnetic field required to demagnetize a material to zero after it has reached *Ms*. Parameters *Mr* and *Hc* are parameters that depend on the size and chemical moiety of the iron-containing substance. *Hc* is expressed as Oersted units (Oe). *Ms* and *Mr* are expressed as electromagnetic units per gram of dried sample (emu/g).

### 2.11. Statistical Analysis

Statistical analyses of BP, HR, and BW gain were performed using a repeated measures ANOVA, with “time” as a repeated factor and “DMF” and “stress” as independent variables. Analyses of vascular functions were performed as concentration–dose curves and analysed using a repeated-measures ANOVA, with “concentration” as a repeated factor, and “DMF” and “stress” as independent variables. A two-way factorial ANOVA (“DMF” and “stress” as independent variables) was used for all other analyses. All ANOVA analyses were followed by Bonferroni’s post hoc test. The normality of the data distribution was tested using the Kolmogorov–Smirnov test. The homogeneity of the data was tested by Levene’s test. Correlations between variables were analysed using Pearson’s correlation coefficient (r). The values were considered to differ significantly when *p* < 0.05. The results are presented as the mean ± standard error of means (SEM). The GraphPad Prism v10.2.1 software (GraphPad Software, Inc., San Diego, CA, USA) and Statistica v13.5 (StatSoft Europe, Hamburg, Germany) were used for the statistical analyses. Analyses were conducted on seven rats per group in the Control and DMF groups and on nine rats per group in the Stress and DMF+Stress groups unless stated otherwise.

### 2.12. Chemicals

All chemicals used in this study were purchased from Merck, Darmstadt, Germany, unless stated otherwise.

## 3. Results

### 3.1. Biometric Parameters

**Figure 1:**
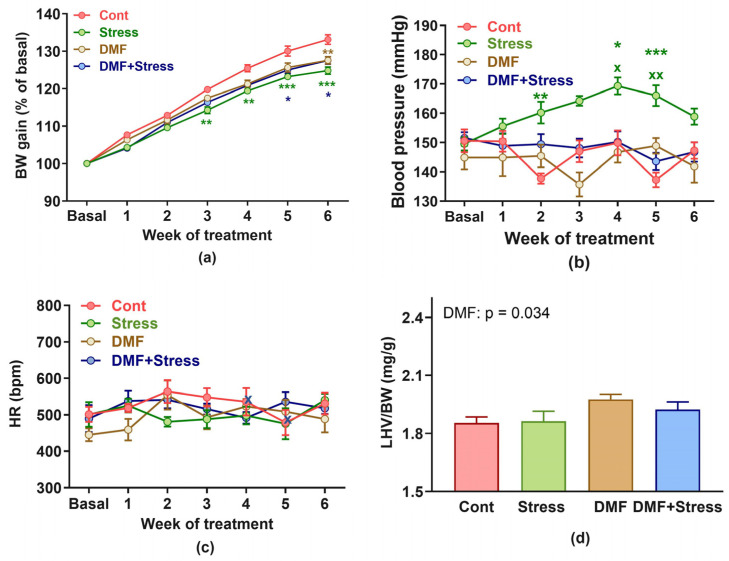
Effect of dimethyl fumarate and stress treatment on body weight gain (**a**), blood pressure (**b**), heart rate, (**c**) and relative left ventricular weight (**d**). Values represent the mean ± SEM. * *p* < 0.05, ** *p* < 0.01, *** *p* < 0.001 vs. Cont group in the respective week of the experiment, ^x^ *p* < 0.05, ^xx^ *p* < 0.001 vs. DMF+Stress group in the respective week of the experiment. Abbreviations: Cont, control; DMF, dimethyl fumarate; BW, body weight; HR, heart rate; bmp, beats per minute; mmHg, millimetres of mercury; LHV, left heart ventricle.

There were no differences in basal BW values between all groups (Cont: 281.71 ± 3.77 g; Stress: 301.66 ± 2.76 g; DMF: 297.71 ± 7.82 g; DMF+Stress: 290.33 ± 2.05 g) at the beginning of the experiment. The ANOVA revealed a significant effect of the stress*DMF*time interaction (F_(6, 168)_ = 7.00, *p* < 0.001) on the relative BW gain during the experiment. Stress significantly (F_(6, 168)_ = 6.00, *p* < 0.001) reduced the BW gain from the third week of the experiment compared to the control group (Figure 1a). In the DMF+Stress group, a significant (*p* < 0.05) reduction in the relative BW gain occurred in the fifth week of the experiment compared to the control group. In addition, DMF itself significantly (*p* < 0.01) reduced the BW gain in the sixth week of the experiment compared to the control group (Figure 1a). An ANOVA revealed a significant effect of the stress*DMF*time interaction (F_(6, 365)_ = 3.84, *p* = 0.001) on BP values. Stress significantly elevated the BP on the second, fourth, and fifth week of the experiment compared to the control group (Figure 1b). The DMF treatment prevented the increase in BP during stress compared to the stress group. There was no effect of stress, DMF, or their combination on HR values during the experiment (Figure 1c). An ANOVA revealed an increase in the LHV/BW ratio in the DMF treatment (F_(1, 28)_ = 4.97, *p* = 0.034) indicating LHV hypertrophy (Figure 1d).

### 3.2. Biochemical Parameters in Plasma

The values of the biochemical plasma parameters and detailed ANOVA results are shown in Table 2.

Plasmatic concentrations of corticosterone (pCort) were elevated in both the Stress and DMF groups vs. Control. The lowest level of pCort was found in the DMF+Stress group (n = 6/group). Considering the main effect, DMF significantly elevated LDH and α-HBDH levels. Parameters that characterise the renal function and plasma electrolytes were not altered by stress or DMF, except for the main effect of stress on albumin concentration (Table 2) in which mild elevation was found.

### 3.3. Gene Expressions

**Figure 2:**
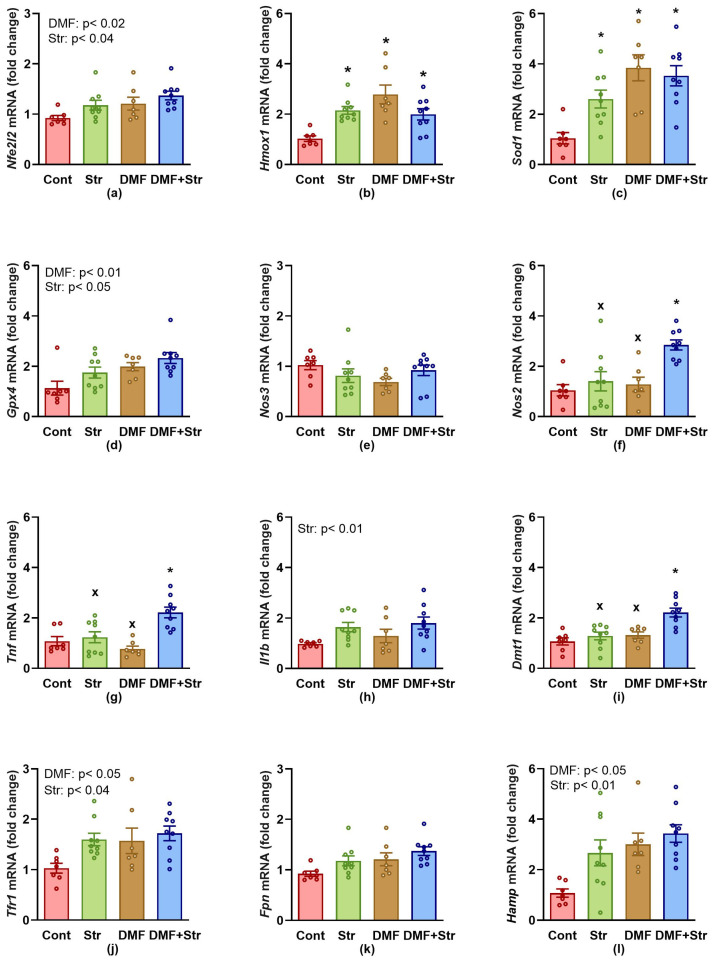
(**a**–**l**) Effect of stress, dimethyl fumarate and their interaction on gene expression in the left heart ventricle. Values represent the mean ± SEM. * *p* < 0.05 vs. Cont group, ^x^ *p* < 0.05 vs. DMF+Stress group. Detailed ANOVA results are in the Supplementary Table S1. Abbreviations: Cont, control group; DMF, dimethyl fumarate; *Nfe2l2*, nuclear factor (erythroid-derived 2)-like 2 gene; *Sod1*, superoxide dismutase 1; *Hmox1*, heme oxygenase 1; *Gpx4*, glutathione peroxidase 4; *Nos2*, inducible nitric oxide synthase; *Nos3*, endothelial nitric oxide synthase; *Tnf*, tumour necrosis factor alpha; *Il1b*, interleukin 1β; *Fpn1*, ferroportin; *Tfr1*, transferrin receptor 1; *Dmt1*, divalent metal ion transporter 1; *Hamp*, hepcidin.

Gene expressions of *Nfe2l2* (Figure 2a), *Hmox1* (Figure 2b), *Sod1* (Figure 2c), *Gpx4* (Figure 2d), *Nos3* (Figure 2e), *Nos2* (Figure 2f), *Tnf* (Figure 2g), *Il1b* (Figure 2h), *Dmt1* (Figure 2i), *Tfr1* (Figure 2j), *Fpn1* (Figure 2k), and *Hamp* (Figure 2l) in the LHV are shown in Figure 2. Both stress and DMF, respectively, significantly up-regulated the expression of *Nfe2l2*, *Gpx4*, *Nos2*, *Tfr1 Dmt1*, and *Hamp* when their main effects were considered. ANOVA revealed a significant main effect of stress on gene expressions for inflammatory mediators *Tfn* and *Il1b.* An ANOVA also revealed a significant effect of stress*DMF interactions in *Sod1*, *Hmox1*, *Nos2*, *Tnf*, and *Dmt1* expressions. Gene expressions of *Fth1* in the LHV were extremely low; therefore, they were not calculated. Detailed ANOVA results are shown in Supplementary Table S1.

There were significant negative correlations between the pCort levels and gene expressions of pro-inflammatory mediators *Tnf* (r = −0.57, *p* = 0.004, n = 24) and *Nos2* (r = −0.53, *p* = 0.008, n = 24) in the LHV.

### 3.4. Nitric Oxide Synthase Activity and Conjugated Dienes Level

**Figure 3:**
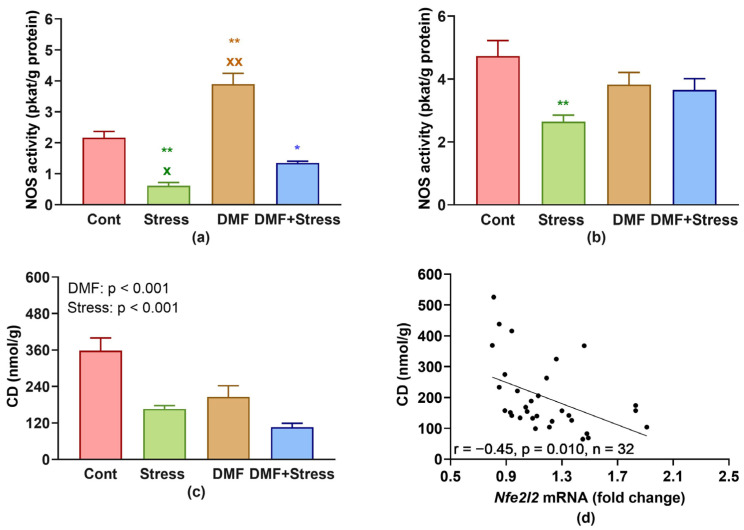
Effect of dimethyl fumarate and stress on total nitric oxide synthase activity in the left heart ventricle (**a**) and in the aorta (**b**), conjugated dienes in the left heart ventricle (**c**), and correlation between gene expression of *Nfe2l2* and level of conjugated dienes (**d**) in the left heart ventricle. * *p* < 0.05, ** *p* < 0.001 vs. Cont group, ^x^ *p* < 0.05, ^xx^ *p* < 0.001 vs. DMF+Stress group. Values represent the mean ± SEM. Abbreviations: DMF, dimethyl fumarate; CD, conjugated dienes; NOS, nitric oxide synthase; *Nfe2l2*, nuclear factor (erythroid-derived 2)-like 2 gene.

In the LHV, ANOVA revealed the significant effect of DMF*Stress interactions (F_(1, 28)_ = 7.05, *p* = 0.013) on the NOS activity in the LHV (Figure 3a). Stress decreased the NOS activity alone (*p* < 0.001) as well as when combined with DMF (*p* = 0.027) vs. the Cont group. However, the NOS activity in the DMF+Stress group was significantly (*p* = 0.037) higher vs. the Stress group. In addition, DMF alone significantly (*p* < 0.001) increased the NOS activity vs. the Cont group (Figure 3a).

In the aorta, the main effect of stress was significant (F_(1, 28)_ = 9.42, *p* = 0.005) (Figure 3b). An ANOVA also revealed the significant effect of DMF*Stress interactions (F_(1, 28)_ = 6.77, *p* = 0.015) on the NOS activity, with significantly reduced NOS activity in the Stress group vs. Cont (*p* = 0.003). In the DMF and DMF+Stress groups, NOS activity did not significantly differ from the Cont group.

Regarding CD in the LHV, the main effect of stress was significant (F_(1, 28)_ = 31.23, *p* < 0.001), and reduced levels of CD were found in stressed groups vs. non-stressed (Figure 3c). Similarly, the main effect of DMF was significant (F_(1, 28)_ = 16.61, *p* < 0.001), and decreased CD levels were found in DMF-treated rats vs. DMF-untreated groups. DMF*Stress interaction was statistically non-significant (F_(1, 28)_ = 3.15, *p* = 0.087).

A correlation analysis showed that CD levels were significantly negatively correlated (r = −0.45, *p* = 0.010, n = 32) with *Nfe2l2* gene expression in the LHV (Figure 3d).

### 3.5. Plasma Iron and Magnetic Properties of Blood and LHV

There were no differences in total iron and ferrous iron concentrations in the plasma of rats among the groups (Table 3).

SQUID magnetometry did not reveal significant differences in *Ms*, *Mr*, and *Hc* (Table 3) among the groups in the LHV (n = 5/group). In blood, significant DMF*Stress interaction was found with the highest *Ms* levels in the DMF group vs. control (Table 3).

There were significant positive correlations between *Ms* in blood and Fe^2+^ in plasma (r = 0.50, *p* = 0.02, n = 20) as well as between *Ms* in blood and *Nfe2l2* expression (r = 0.48, *p* = 0.04, n = 20), *Hmox1* (r = 0.71, *p* < 0.0001, n = 20), *Sod1* (r = 0.62, *p* = 0.003, n = 20), and *Gpx4* (r = 0.50, *p* = 0.02, n = 20) in the LHV.

### 3.6. Vascular Reactivity

KPSS-induced contraction in the Cont (20.27 ± 1.04 mN), Stress (20.69 ± 1.26 mN), DMF (22.76 ± 1.48 mN), and DMF+Stress (22.44 ± 2.29 mN) groups did not differ among the groups (DMF*Stress interaction: F_(1, 24)_ = 0.055, *p* = 0.816).

**Figure 4:**
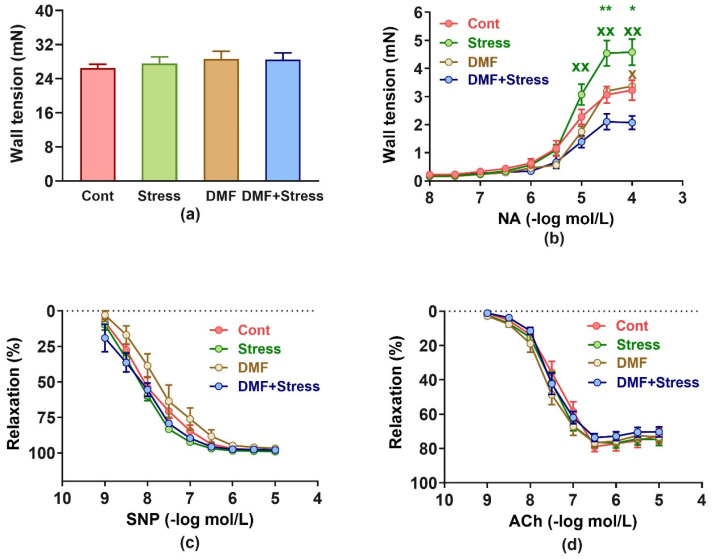
Effect of dimethyl fumarate and stress on serotonin-induced (**a**) and noradrenalin-induced contractions (**b**) and on sodium nitroprusside-induced (**c**) and acetylcholine-induced (**d**) relaxations. Values represent the mean ± SEM. * *p* < 0.01, ** *p* < 0.001 vs. Cont group at a respective concentration, ^xx^ *p* < 0.001 vs. DMF+Stress group at the respective concentrations. Symbols are always shown in the colour of the corresponding experimental group. Abbreviations: DMF, dimethyl fumarate; NA, noradrenaline; SNP, sodium nitroprusside; ACh, acetylcholine.

Similarly, serotonin-induced contractions (Figure 4a) were unchanged among the groups (DMF*Stress interaction: F_(1, 24)_ = 0.18, *p* = 0.674). For NA-induced contractions, there was a significant DMF*Stress*NA interaction (F_(1, 192)_ = 15.45, *p* = 0.0001). DMF did not alter NA-induced contractions vs. the Cont and DMF groups (Figure 4b). Stress significantly elevated NA-induced contractions at concentrations of 5 × 10^−5^ mol/L (*p* < 0.001) and 10^−4^ mol/L (*p* = 0.003) compared to the Cont group. In the DMF+Stress group, DMF significantly suppressed the stress-induced contraction concentrations at the NA concentrations of 10^−5^–10^−4^ mol/L vs. the Stress group (*p* < 0.001 at each of the abovementioned concentrations). In addition, at the maximal NA concentration, a significant (*p* = 0.036) decrease in contraction was found in the DMF+Stress group compared to the DMF group (Figure 3b).

The endothelium-independent relaxation induced by SNP (DMF*Stress*SNP interaction concentration: F_(1, 192)_ = 0.71, *p* = 0.684, Figure 4c) and endothelium-dependent relaxation induced by ACh (DMF*Stress*ACh interaction concentration: F_(1, 192)_ = 0.65, *p* = 0.735, Figure 4d) did not differ significantly in all DMF, Stress, and DMF+Stress groups compared to the control group.

## 4. Discussion

In this study, we used 10-week-old BHR males as an experimental model of rats with a genetic predisposition to elevated blood pressure to determine the effects of DMF, stress, and their interactions. The main findings of this study are as follows: (1) The chronic social stress produced by crowding for six weeks significantly increased BP and plasma corticosterone while reducing BW gain. We also found elevated *Nfe2l2* gene expression in the hearts of crowding stress-exposed rats. (2) DMF alone did not affect BP and vascular functions in the femoral artery. However, DMF elevated the *Nfe2l2* gene expression in the hearts, which was associated with the development of LHV hypertrophy. (3) Our results also showed that DMF prevented stress-induced BP and corticosterone increases. This was associated with a reduction in NA-induced contractions in the femoral artery on the one hand and an increase in the gene expression of inflammatory mediators (*Tnf*, *Nos2*) in the LHV on the other hand.

The role of chronic stress in the development of CVDs is known; however, the development of hypertension may depend on several factors, such as age, sex, and genetic predisposition [19,23]. As written above, BHRs are more sensitive to stress than normotensive rats, making them suitable for investigating the effects of stress and the antihypertensive action of various substances. In BHR, an increase in crowding stress-induced BP was also seen in our previous studies in young and adult rats [41,42], and the ability of phytochemicals to decrease BP in this rat strain was demonstrated using flavanol (-)-epicatechin [42].

The preventive effect of DMF on a stress-induced BP increase was previously found by Jimenez et al. [43] in mice exposed to noise over a 4-day period, as well as in studies that used models of hypertension induced by co-treatment with dexamethasone and a high-fat diet in rats [44,45]. However, our study is the first to describe the preventive effects of DMF treatment on chronic social stress-induced hypertension. Herein, we found that DMF alone failed to reduce BP in control conditions, which is in agreement with previous findings in mice [43]. We also found that DMF prevented the stress-induced decrease in BW gain vs. stress-exposed rats, despite DMF alone also reducing the BW gain at the end of the experiment vs. the control group. The BW gain reduction in DMF-treated rats may be related to the increased pCort levels found in this group, which may result from hyperactivation of the hypothalamic–pituitary–adrenal axis by upregulating adrenocorticotrophic hormone receptors in the adrenal glands and downregulating glucocorticoid receptors in the pituitary, as found in a study by Prevatto et al. [46].

Interestingly, we found that the main effect of DMF on the LHV/BW ratio was significant, suggesting an impact on the heart structure and the development of LHV hypertrophy. In general, LHV hypertrophy results from hypertension [47] resulting from altered kidney function or reduced NO production [48], specifically from the lack of NO produced by endothelial NOS (eNOS) [49,50]. Since, in this study, DMF did not alter BP, plasma markers of kidney damage, or *Nos3* gene expression and did not reduce NOS activities in the LHV under control conditions, LHV hypertrophy observed in DMF-treated rats must be attributed to other mechanisms. An analysis of plasma biochemical markers showed that DMF (assessed as a main effect) increased total LDH and α-HBDH activities (α-HBDH reflects the activity of LDH1 and LDH2 isozymes), which are mainly distributed in the heart [51]. Although these enzymes are not specific to cardiac disorders, elevated LDH and α-HBDH, together with the observed increase in LHV/BW ratio, suggest the effect of prolonged DMF treatment on the heart structure. Thus, we assume that LHV hypertrophy in the DMF-treated rats could result from reductive stress due to the continuous overactivation of NRF2-regulated antioxidant enzymes. We found an elevated expression of *Nfe2l2* (as the main effect of DMF) and NRF2-regulated genes encoding antioxidant enzymes (*Sod1*, *Hmox1* vs. control group, *Gpx4* as the main effect) as well as reduced CD content below the control level, which negatively correlated with the expression of *Nfe2l2*. This finding agrees with studies that used transgenic mice in which a role for NRF2 in the development of reductive stress associated with cardiac hypertrophy was found [52,53,54].

Regarding crowding stress itself, despite elevating BP, it failed to alter the LHV/BW ratio or the LDH and α-HBDH activities vs. control, and it failed to increase the plasma markers of renal damage over their physiological ranges. The fact that crowding stress did not lead to LHV hypertrophy may be related to the cardiovascular phenotype of BHRs because mild LHV hypertrophy was also present in control BHRs compared with normotensive rats [41]. Thus, the hearts of BHRs may be, at least partially, adapted to the additional pressure overload induced by the relatively mild stressor. In addition, crowding stress elevated the expressions of *Nfe2l2* (as the main effect) and the antioxidant genes *Hmox1*, *Sod1*, and *Gpx4*, and it reduced CD levels similarly to DMF. On the other hand, stress, in contrast to DMF, reduced the total NOS activity in the LHV when acting alone or together with DMF, proving an important role of stress in the modulation of NO release via mechanisms that were reviewed previously [19]. Moreover, despite the elevated *Nfe2l2* expression, indicating the inhibition of the release of pro-inflammatory mediators [9], the expressions of genes encoding inflammatory mediators (*Tnf*, *Nos2*, *Il1b*) and inflammatory marker hepcidin (*Hamp*) were elevated in crowding-stress-exposed rats (considered as the main effects in all abovementioned genes), proving the pro-inflammatory influence of chronic stress, as found in preclinical and clinical studies [55,56,57]. Chronic stress is linked to inflammation through imbalances in the autonomic nervous system and stress hormone release. Specifically, the long-term elevation of catecholamines and cortisol/corticosterone is associated with the release of pro-inflammatory factors such as nuclear factor kappa-light-chain-enhancer of activated B cells (NFκB). Additionally, a decrease in parasympathetic activity, marked by vagal withdrawal, may contribute to increased production of pro-inflammatory cytokines IL-1β and TNF-α [57,58] followed by the increase in inducible NOS gene expression [58], the outcomes that were present also in this study. Our results thus showed that despite the activation of NRF2 function during chronic social stress, considered a protective mechanism, this mechanism was insufficient to reverse chronic stress-induced pro-inflammatory processes in the hearts of BHRs.

To determine if the simultaneous administration of DMF during chronic stress can reverse stress-induced disorders, we investigated how the interaction of both factors affects the expression of *Nfe2l2* and selected NRF2 target genes. Interestingly, despite the prevention of a BP increase, the co-treatment of stressed rats with DMF did not lead to a significant upregulation of the gene expression of *Nfe2l2* or the abovementioned antioxidant genes when compared with DMF or stress acting alone. However, we found a significant upregulation of the pro-inflammatory genes *Tnf* and *Nos2* in the DMF+stress group. These findings were, in fact, opposite to what was hypothesised, suggesting negative consequences of long-term DMF treatment in systemic stress conditions. The mechanisms of such an effect are unclear, but the study of Barret et al. [59] showed that chronic stress-mediated priming alters inflammatory responses in mice and humans towards a hyperinflammatory phenotype. An increased expression of *Tnf* and *Nos2* can also result from lower pCort levels in the DMF+Stress group due to the lack of anti-inflammatory action of corticosterone [60], independently of NFR2-dependent mechanisms. However, in contrast to our findings, Amin et al. found a reduction in pro-inflammatory markers if DMF was administered to rats with experimental diabetes [61].

It is well known that the vascular bed plays a critical role in BP regulation, not only in control conditions but also in various diseased states and systemic stress. During systemic stress, changes in the functional state of arteries depend on many factors, such as animal strain, sex, age, type of stressor, and its duration [19], as well as the type of arteries (conduit, resistance) and the presence of various receptors in the given artery [62]. Thus, depending on the situation, stress can induce vasorelaxation or vasoconstriction, and these mechanisms have been discussed in previous studies [19,63,64]. In this study, we found that 6-week crowding did not affect the endothelium-dependent NO-mediated ACh-induced relaxation or endothelium-independent NO-mediated SNP-induced relaxations in the femoral arteries of BHRs. However, stress significantly increased NA-induced constriction, which was associated with a reduction in NOS activity and an increase in BP.

Regarding DMF, its effect on vascular function is not precisely known. Available studies have consistently shown that DMF has no significant effect on SNP-induced relaxation [43,61], which was also found in this study. The aforementioned studies [43,61] as well as this study also found that DMF has no effect on ACh-induced relaxation under control conditions. These findings were independent of the artery type and the experimental model.

However, differences were found regarding the ability of DMF to alter ACh-induced relaxation in stress or diseased states. Jimenez et al. in noise-exposed mice [43] and Amin et al. [61] in rats with streptozotocin-induced diabetes used the aorta, in which endothelial dysfunction (determined as reduced ACh-induced relaxation) was developed in the respective experimental model, while DMF treatment restored endothelial function via a reduction in oxidative stress. These results show that DMF may prevent ROS-induced endothelial dysfunction. However, in our model of crowding stress-exposed BHRs, endothelial dysfunction (i.e., reduced ACh-induced relaxation) was not found in the femoral artery, and thus, its improvement cannot be expected. It might be because crowding stress did not produce oxidative stress in BHRs, judging by the reduced level of CD in the heart.

Regarding contractile function, all studies [43,61], and this one, have consistently shown that DMF does not alter KCl-induced contractions under control conditions. Jimenez et al. showed no effect of DMF on prostaglandin F2-α-induced contractions in any group investigated [43]. Amin et al. investigated phenylephrine-induced contractions, which were not affected by DMF in control, but they were reduced under diabetic conditions [61].

We focused on serotonin- and NA-induced contractions. There was no effect of DMF or stress on serotonin-induced contractions. However, NA-induced contraction, which is more relevant to be investigated in stress conditions, was elevated in stressed rats, proving the activation of the sympatho-adrenal system [65] during chronic crowding. In DMF-treated rats, in agreement with an unchanged BP, NA-induced contractions were unchanged (vs. the control group). However, DMF significantly reduced NA-induced contractions in stress-exposed rats (vs. stress alone). It can partially result from DMF-induced prevention in reducing NOS activity in the DMF+Stress group as NO acts against the sympathetic activation and NA-induced contractions [19,66]. In addition, the reduced pCort levels in this group can also contribute to weakened NA-induced contractions, as it was shown that glucocorticoids can potentiate α-1 adrenergic receptor-mediated contractions [67,68]. We assume that the DMF-induced decrease in NA-induced contractions is the primary mechanism by which DMF prevented stress-induced hypertension in the crowding stress model. However, because of the complex interplay among the stress-induced central and peripheral alterations and the DMF-induced mechanisms, the exact mechanism of a DMF-associated reduction in NA-induced constriction remains to be elucidated in a specifically designed study.

The important contribution of our study is new knowledge of the NRF2 effect on the expression of genes involved in iron metabolism in the LHV in conditions of DMF treatment, chronic stress, and their interaction, as it is known that optimal iron concentration is necessary for normal heart function and structure [69]. In plasma, no differences in total or divalent iron concentrations were found in this study. However, plasma iron concentrations do not reflect the iron stores in the individual tissues, and the biochemical determination of iron may not be sensitive enough to detect small changes in iron concentration in biological samples. Thus, we used SQUID magnetometry to determine magnetic parameters such as *Ms*, *Mr*, and *Hc*, which characterise the alterations of mainly iron-containing compounds in the tissues and blood [40]. In blood, SQUID magnetometry revealed significantly elevated *Ms* in the DMF group. In addition, *Ms* in blood correlated positively with plasma Fe^2+^ levels and with *Nfe2l2*, *Hmox1*, *Sod1*, and *Gpx4* gene expressions in the LHV.

However, in the LHV, despite no alterations in magnetic parameters in all groups investigated vs. the control group, we found upregulated gene expressions of genes involved in iron metabolism: *Tfr1* (the receptor for transferrin-bound iron entry into cells, its elevated expression serves as a tissue iron deficiency marker), *Dmt1* (the major divalent iron transporter contributing to divalent iron uptake in most types of cells), and *Hamp* (the gene encoding hepcidin, a hormone that inhibits iron exporter ferroportin function) determined as the main effects of stress and DMF, respectively. Regarding hepcidin, its release, mainly from the liver, is elevated during inflammation. In the heart, elevated *Hamp* expression may result mainly from hypoxia or iron deficiency in cells [70]. Of these conditions, based on the mechanism of transcription regulation of intracellular iron homeostasis [10], our data would suggest decreased iron levels in the hearts. Interestingly, the results obtained in the DMF and stress groups, respectively, were similar in terms of the expressions of genes involved in iron metabolism. This may be because we found comparable levels of pCort in both groups, which in turn similarly affected the expression of genes in iron metabolism. Briefly, elevated levels of pCort can increase the mobilisation of iron from the tissues and elevated excretion of iron through urine [70]. These alterations may have a significant impact on iron homeostasis in all organs and may have implications for health during long-term DMF treatment or chronic stress exposure, independently of the presence of the second factor.

Like any experimental study, this study also has its limitations. We used a stress-sensitive rat model (offspring of hypertensive dams and normotensive sires) and we included only males; thus, it is unknown whether observed changes, mainly in NFR2-regulated gene expressions during chronic DMF treatment, are translatable to BHR females or other mammals and even humans exposed to different stressors. In addition, BHRs are an outbred strain, and thus, the results might slightly differ among the studies despite their sensitivity to stress has been confirmed in several studies. Finally, we did not determine the protein expressions of genes investigated in this study; instead, we focused on gene expressions in association with selected biochemical and physiological outcomes. However, understanding complex interactions and molecular mechanisms needs further specific investigations, not only in the heart but also in other organs.

## 5. Conclusions

In conclusion, this study is the first to show that the NRF2 activator, DMF, prevents chronic stress-induced hypertension by reducing NA-induced contractions in the femoral arteries. However, our results also show that long-term DMF administration may alter iron metabolism in the heart and produce reductive stress manifested in LHV hypertrophy. In addition, we found that chronic DMF treatment may increase the expression of genes of inflammatory factors when combined with chronic stress. This indicates a need for the careful consideration of long-term DMF treatment considering its impact on the heart.

## Figures and Tables

**Table 1:** Primers used in the RT-qPCR.

Gene	Forward Primer	Reverse Primer	Tm (°C)	Amplicon Size (bp)
*Nfe2l2* (NM_031789.2)	TGCCATTAGT CAGTCGCTCTC	ACCGTGCCT TCAGTGTGC	60	102
*Hmox1* (NM_017165.4)	AGAAGAGG CTAAGACCGCCT	TCTGGTCTTTG TGTTCCTCTG TC	60	86
*Sod1* (NM_017050.1)	CTGAAGGCGA GCATGGGTTC	TCCAACATGCC TCTCTTCATC C	60	131
*Gpx4* (NM_017165.4)	TAAGTACAGGG GTTGCGTGTG	CAAGGGAAGG CCAGGATT CG	60	135
*Fpn1* (NM_133315.2)	GACCTCACCTAA AGATACTGAGCC	GAAGGGTTCTG CGATCTGGG	59	130
*Tfr1* (NM_022712.1)	GCTATGAGGAA CCAGACCGC	CACTGGACTTC GCAACACCA	58	78
*Dmt1* (NM_013173.2)	CTACTTGGGT TGGCAGTGTTTG	ATCTTCGCTCA GCAG GAC TTT	60	94
*Fth1* (NM_012848.2)	GACCTCACCTA AAGATACTGAGCC	GAAGGGTTCT GCGATCTGGG	59	130
*Hamp* (NM_053469.1)	CTATCTCCGG CAACAGACGAG	TGTCTCGCTTC CTTCGCTTC	60	110
*Nos2* (NM_012611.3)	AAACGCTACA CTTCCAACGC	TGCTGAGAGC TTTGTTGAGGTC	59	91
*Nos3* (NM_021838.2)	GATCCCCCG GAGAATGGAGA	TCGGATTTTGT AACTCTTGTGCT	60	105
*Tnf* (NM_012675.3)	CGTCAGCCGAT TTGCCATTTC	TGGGCTCATAC CAGGGCTT	60	116
*Il1b* (NM_031512.2)	CACCTCTCAAGC AGAGCACAG	GGGTTCCATGG TGAA GTCAAC	60	79
*Actb* (NM_031144.3)	CTCTGTGTGGA TTGGTGGCT	CGCAGCTCAGT AACAGTCCG	59	139

Abbreviations: *Nfe2l2*, nuclear factor (erythroid-derived 2)-like 2 gene; *Hmox1*, heme oxygenase 1; *Sod1*, superoxide dismutase; *Gpx4*, glutathione peroxidase 4; *Fpn1*, ferroportin; *Tfr1*, transferrin receptor 1; *Dmt1*, divalent metal ion transporter 1; *Fth1*, Ferritin heavy chain 1; *Hamp*, hepcidin; *Nos2*, inducible nitric oxide synthase; *Nos3*, endothelial nitric oxide synthase; *Tnf*, tumour necrosis factor alpha; *Il1b*, interleukin 1β; *Actb*, β-actin.

**Table 2:** Effect of DMF and stress on biochemical parameters in plasma.

Parameter	Group				ANOVA Results		
	Control	Stress	DMF	DMF+Stress	Stress	DMF	Interaction
**pCort** (ng/mL)	20.50 ± 2. 9	41.2 ± 3.9 *^,x^	43.3 ± 4.7 *^,x^	12.39 ± 1.65	F_(1, 20)_ = 2.12 *p* = 0.161	F_(1, 20)_ = 0.74 *p* = 0.399	**F_(1, 20)_ = 54.28** ***p* < 0.0001**
**LDH** (U/L)	500.0 ± 30.8	562.3 ± 59.2	688.0 ± 57.2	656.1 ± 50.9	F_(1, 28)_ = 0.08 *p* = 0.775	**F_(1, 28)_ = 7.11** ***p* = 0.013**	F_(1, 28)_ = 0.79 *p* = 0.380
**α-HBDH** (U/L)	121.3 ± 9.0	142.9 ± 18.3	176.0 ± 16.2	183.8 ± 24.1	F_(1, 28)_ = 0.59 *p* = 0.448	**F_(1, 28)_ = 6.26** ***p* = 0.018**	F_(1, 28)_ = 0.13 *p* = 0.720
**CRE** (U/L)	48.3 ± 3.3	47.9 ± 1.6	49.0 ± 0.9	51.7 ± 1.5	F_(1, 28)_ = 0.33 *p* = 0.568	F_(1, 28)_ = 1.31 *p* = 0.262	F_(1_, _28)_ = 0.61 *p* = 0.442
**TP** (U/L)	69.5 ± 0.6	71.3 ± 0.8	70.1 ± 0.9	71.2 ± 0.7	F_(1, 28)_ = 3.48 *p* = 0.073	F_(1, 28)_ = 0.09 *p* = 0.772	F_(1, 28)_ = 0.22 *p* = 0.646
**ALB** (U/L)	40.0 ± 0.3	41.0 ± 0.2	40.1 ± 0.7	41.0 ± 0.4	**F_(1, 28)_ = 4.95** ***p* = 0.034**	F_(1, 28)_ = 0.02 *p* = 0.878	F_(1, 28)_ 0.02 *p* = 0.878
**GLO** (U/L)	29.5 ± 0.4	30.3 ± 0.7	29.9 ± 0.7	30.2 ± 0.5	F_(1, 28)_ = 0.66 *p* = 0.424	F_(1, 28)_ = 0.06 *p* = 0.802	F_(1, 28)_ = 0.21 *p* = 0.648
**Urea** (U/L)	6.2 ± 0.2	6.2 ± 0.2	6.2 ± 0.2	6.5 ± 0.2	F_(1, 28)_ = 0.67 *p* = 0.421	F_(1, 28)_ = 0.58 *p* = 0.453	F_(1, 28)_ = 0.71 *p* = 0.407
**Na^+^** (μmol/L)	152.7 ± 1.7	150.9 ± 0.5	151.6 ± 1.1	150.0 ± 1.2	F_(1, 28)_ = 0.67 *p* = 0.420	F_(1, 28)_ = 1.92 *p* = 0.177	F_(1, 28)_ = 0.01 *p* = 0.903
**K^+^** (μmol/L)	5.9 ± 0.1	6.1 ± 0.1	5.9 ± 0.1	6.1 ± 0.1	F_(1, 28)_ = 3.4 *p* = 0.076	F_(1, 28)_ = 0.01 *p* = 0.911	F_(1, 28)_ = 0.14 *p* = 0.712
**Cl^−^** (μmol/L)	105.0 ± 2.9	107.2 ± 0.5	106.4 ± 1.0	106.9 ± 0.7	F_(1, 28)_ = 0.88 *p* = 0.357	F_(1, 28)_ = 0.14 *p* = 0.711	F_(1, 28)_ = 0.37 *p* = 0.547
**CO_2_** (μmol/L)	27.7 ± 0.2	27.7 ± 0.2	28.2 ± 0.4	27.6 ± 0.5	F_(1, 28)_ = 0.77 *p* = 0.387	F_(1, 28)_ = 0.54 *p* = 0.468	F_(1, 28)_ = 0.69 *p* = 0.412

Values represent the mean ± SEM. * *p* < 0.05 vs. Control group, ^x^ *p* < 0.05 vs. DMF+Stress group. Abbreviations: DMF, dimethyl fumarate; pCort, plasma corticosterone; LDH, lactate dehydrogenase; α-HBDH, α-hydroxybutyrate dehydrogenase; CRE, creatinine; TP, total protein; ALB, albumin; GLO, globulin.

**Table 3:** Effect of dimethyl fumarate and stress on plasma iron concentrations and magnetic parameters *Ms*, *Mr*, and *Hc* in blood and tissue of the left heart ventricle.

Parameter	Group				ANOVA Results		
	Control	Stress	DMF	DMF+Stress	Stress	DMF	Interaction
**Iron in plasma**							
**Total Fe** (µmol/L)	65.5 ± 5.1	74.7 ± 9.2	89.5 ± 12.6	66.1 ± 8.8	F_(1, 28)_ = 0.59 *p* = 0.447	F_(1, 28)_ = 0.65 *p* = 0.425	F_(1, 28)_ = 3.01 *p* = 0.093
**Fe^2+^** (µmol/L)	37.5 ± 2.9	51.2 ± 6.4	60.8 ± 8.9	51.1 ± 8.6	F_(1, 28)_ = 0.07 *p* = 0.790	F_(1, 28)_ = 2.43 *p* = 0.130	F_(1, 28)_ = 2.49 *p* = 0.125
**SQUID magnetometry in blood**							
***Ms*** (memu/g)	171.4 ± 2.64	188.5 ± 8.22	203.0 ± 3.88*	184.0 ± 6.11	F_(1, 16)_ = 0.03 *p* = 0.88	**F_(1, 16)_ = 5.76 ** ***p* = 0.028**	**F_(1, 16)_ = 10.28** ***p* = 0.006**
***Mr*** (memu/g)	0.15 ± 0.008	0.15 ± 0.006	0.16 ± 0.004	0.14 ± 0.009	F_(1, 16)_ = 2.18 *p* = 0.159	F_(1, 16)_ = 0.01 *p* = 0.945	F_(1, 16)_ = 3.62 *p* = 0.075
***Hc*** (Oe)	16.5 ± 1.04	15.7 ± 0.83	16.7 ± 0.56	15.12 ± 1.16	F_(1, 16)_ = 1.55 *p* = 0.231	F_(1, 16)_ = 0.04 *p* = 0.849	F_(1, 16)_ = 0.20 *p* = 0.658
**SQUID magnetometry in the left heart ventricle**							
***Ms*** (memu/g)	35.17 ± 1.38	34.17 ± 1.16	34.95 ± 2.18	33.75 ± 1.15	F_(1, 28)_ = 0.56 *p* = 0.462	F_(1, 28)_ = 0.05 *p* = 0.827	F_(1, 28)_ = 0.01 *p* = 0.946
***Mr*** (μemu/g)	33 ± 7	33 ± 6	27 ± 4	21 ± 5	F_(1, 28)_ = 0.31 *p* = 0.581	F_(1, 28)_ = 2.62 *p* = 0.117	F_(1, 28)_ = 0.29 *p* = 0.589
***Hc*** (Oe)	14.85 ± 4.27	18.33 ± 3.17	17.71 ± 2.59	10.33 ± 2.90	F_(1, 28)_ = 0.03 *p* = 0.868	F_(1, 28)_ = 2.38 *p* = 0.134	F_(1, 28)_ = 0.79 *p* = 0.381

Values represent the mean ± SEM. Abbreviations: DMF, dimethyl fumarate; SQUID, superconducting quantum interference device; *Ms*, saturation magnetization; *Mr*, remanent magnetization; *Hc*, coercivity; Oe, Oersted unit of magnetic field strength; memu, milielectromagnetic unit; μemu, microelectromagnetic unit.

## Data Availability

The original contributions presented in this study are included in the article and Supplementary Materials; further inquiries can be directed to the corresponding author.

## Supplementary Materials

The following supporting information can be downloaded at: https://www.mdpi.com/article/10.3390/antiox13080947/s1, Detailed ANOVA results of gene expressions in the heart are in the Supplementary Table S1.
