# Supplementary material for: Dimethyl Fumarate Prevents the Development of Chronic Social Stress-Induced Hypertension in Borderline Hypertensive Rats
Source: Antioxidants (Basel). 2024 Aug 3;13(8):947. doi: 10.3390/antiox13080947 (PMC11351876; doi:10.3390/antiox13080947)
Supplement: Supplementary file 1 [file antioxidants-13-00947-s001.zip › antioxidants-3115075-supplementary.pdf]

Dimethyl Fumarate Prevents the Development of Chronic Social Stress-Induced Hypertension in Borderline Hypertensive Rats

Michal Kluknavsky [1], Peter Balis [1], Silvia Liskova [1,2], Andrea Micurova [1], Martin Skratek [3], Jan Manka [3] and Iveta Bernatova [1,*]

Supplementary Table S1. ANOVA results of the effect of stress and DMF on genes expressions in the left heart ventricle.

Gene	Group				ANOVA results		
	Control	Stress	DMF	DMF + Stress	Stress	DMF	Interaction
Nfe2l2	0.92 ± 0.05	1.18 ± 0.10	1.21 ± 0.13	1.37 ± 0.09	**$F_{(1,28)}$ = 4.82 p = 0.037**	**$F_{(1,28)}$ = 6.29 p = 0.018**	$F_{(1,28)}$ = 0.25 p = 0.623
Sod1	1.03 ± 0.12	2.60 ± 0.35*	3.84 ± 0.52*	3.53 ± 0.40*	$F_{(1,28)}$ = 2.69 p = 0.112	**$F_{(1,28)}$ = 23.89 p < 0.001**	**$F_{(1,28)}$ = 6.10 p = 0.020**
Hmox1	1.03 ± 0.11	2.15 ± 0.15*	2.78 ± 0.37*	1.99 ± 0.23*	$F_{(1,28)}$ = 0.51 p = 0.479	**$F_{(1,28)}$ = 12.08 p = 0.002**	**$F_{(1,28)}$=17.37 p < 0.001**
Gpx4	1.13 ± 0.28	1.75 ± 0.22	1.99 ± 0.16	2.33 ± 0.22	**$F_{(1,28)}$ = 4.61 p = 0.041**	**$F_{(1,28)}$ = 10.2 p = 0.003**	$F_{(1,28)}$ = 0.38 p = 0.541
Nos2	1.05 ± 0.22	1.41 ± 0.38^x	1.28 ± 0.29^x	2.85 ± 0.20*	**$F_{(1,28)}$ = 10.75 p = 0.003**	**$F_{(1,28)}$ = 8.14 p = 0.008**	**$F_{(1,28)}$ = 4.23 p = 0.049**
Nos3	1.02 ± 0.09	0.81 ± 0.14	0.69 ± 0.07	0.92 ± 0.10	$F_{(1,28)}$ = 0.01 p = 0.926	$F_{(1,28)}$ = 1.08 p = 0.308	$F_{(1,28)}$ = 4.04 p = 0.054
Tnf	1.08 ± 0.18	1.23 ± 0.22^x	0.77 ± 0.11^x	2.22 ± 0.21*	**$F_{(1,28)}$ = 16.57 p < 0.001**	$F_{(1,28)}$ = 2.98 p = 0.096	**$F_{(1,28)}$=10.84 p = 0.003**
Il1b	0.98 ± 0.04	1.62 ± 0.20	1.20 ± 0.24	1.85 ± 0.14	**$F_{(1,28)}$ = 13.11 p = 0.001**	$F_{(1,28)}$ = 1.63 p = 0.213	$F_{(1,28)}$=0.001 p = 0.971
Fpn1	1.06 ± 0.11	1.29 ± 0.18	1.59 ±0.20	1.24 ± 0.16	$F_{(1,28)}$ = 0.11 p = 0.741	$F_{(1,28)}$ = 1.95 p = 0.173	$F_{(1,28)}$ = 2.90 p = 0.100
Tfr1	1.03 ± 0.10	1.60 ± 0.12	1.57 ± 0.25	1.72 ± 0.14	**$F_{(1,28)}$ = 4.96 p = 0.034**	**$F_{(1,28)}$ = 4.27 p = 0.048**	$F_{(1,28)}$ = 1.71 p = 0.202
Dmt1	1.07 ± 0.14	1.29 ± 0.15^x	1.32 ± 0.12^x	2.22 ± 0.17*	**$F_{(1,28)}$ = 12.74 p = 0.001**	**$F_{(1,28)}$ = 14.47 p = 0.001**	**$F_{(1,28)}$ = 4.77 p = 0.038**
Hamp	1.07 ± 0.16	2.66 ± 0.51	3.00 ± 0.44	3.43 ± 0.35	**$F_{(1,28)}$ = 6.08 p = 0.020**	**$F_{(1,28)}$ = 10.81 p = 0.003**	$F_{(1,28)}$ = 2.01 p = 0.166

Values represent the mean ± SEM. *p < 0.05 vs. Cont group, ^{x}p < 0.05 vs. DMF+Stress group. Abbreviation: DMF, dimethyl fumarate; *Nfe2l2*, nuclear factor (erythroid-derived 2)-like 2 gene; *Sod1*, superoxide dismutase 1; *Hmox1*, heme oxygenase 1; *Gpx4*, glutathione peroxidase 4; *Nos2*, inducible nitric oxide synthase; *Nos3*, endothelial nitric oxide synthase; *Tnf*, tumour necrosis factor alpha; *Il1b*, interleukin 1β; *Fpn1*, ferroportin; *Tfr1*, transferrin receptor 1; *Dmt1*, divalent metal ion transporter 1; *Hamp*, hepcidin
